# Tolerance and Persister Formation in Oral Streptococci

**DOI:** 10.3390/antibiotics9040167

**Published:** 2020-04-08

**Authors:** Stephanie Suppiger, Monika Astasov-Frauenhoffer, Irene Schweizer, Tuomas Waltimo, Eva M. Kulik

**Affiliations:** 1 Department of Oral Health & Medicine, University Center for Dental Medicine, University of Basel, 4058 Basel, Switzerland; stephanie.suppiger@unibas.ch (S.S.); tuomas.waltimo@unibas.ch (T.W.); 2 Department Research, University Center for Dental Medicine, University of Basel, 4058 Basel, Switzerland; m.astasov-frauenhoffer@unibas.ch (M.A.-F.); irene.m.schweizer@bluewin.ch (I.S.)

**Keywords:** streptococci, chlorhexidine, tolerance, persister

## Abstract

The aim of this study was to analyze the potential influence of long-term exposure in subinhibitory concentrations of chlorhexidine on the emergence of tolerant and/or persistent cells in oral streptococci. The two oral streptococcal isolates *S. mutans* ATCC25175 and *S. sobrinus* ATCC33402 were incubated, after long-term subinhibitory exposure to chlorhexidine, in liquid growth media containing high concentrations of chlorhexidine. A distinct subpopulation of more chlorhexidine-tolerant cells could be detected in streptococci that had been previously exposed to subinhibitory concentrations of chlorhexidine but not in the control strains. These more biocide-tolerant and persisting microbial subpopulations might also arise in vivo. Therefore, the rational and proper use of antimicrobials in dentistry, especially when used over a long period of time, is crucial.

## 1. Introduction

The oral cavity harbors a complex and diverse microbial community where oral microorganisms are organized in biofilms, complex structures that will adhere on various oral surfaces. When organized in such biofilms, microorganisms are able to adapt to changes in their environment. Additionally, the biofilm structure protects the microorganisms from antibiotics as well as from biocides such as chlorhexidine (CHX). Pioneer bacterial species, mostly oral streptococci, adhere to the salivary pellicle on teeth forming an early oral biofilm. Secondary colonization by other microorganisms to these bacteria, mediated by adhesins, will follow resulting in a mature plaque that may lead to oral diseases such as caries or periodontitis [1,2,3].

Dental caries is a highly prevalent oral disease affecting both children and adults [4,5]. The main etiological factor, a cariogenic microbiota that metabolizes dietary carbohydrates into acids, has long been known. Nevertheless, caries still remains a major health problem globally [6]. Various models exist which try to explain the transition from a health-compatible oral microbiota to a cariogenic one. Today, it is accepted that caries is a multifactorial disease where three main factors, the presence of acidogenic and acidophilic microorganisms, a diet containing sugar, and host factors such as salivary flow or oral hygiene, will lead to a dysbiotic state. Although several bacterial species have been associated with dental caries, mutans streptococci, i.e., *Streptococcus mutans* and *Streptococcus sobrinus,* have been implicated as the primary causative agents. One of the most important virulence factors of *S. mutans* is its ability to form biofilms by synthesizing glucans from sucrose which enables bacterial cells to firmly adhere to tooth surfaces [7].

Periodontitis is an inflammatory disease of the periodontium that may eventually lead to tooth loss. Worldwide, it affects up to 60% of the population [8,9]. There appears to be an association of periodontitis with certain systemic diseases such as cardiovascular disease, complications in pregnancy or Alzheimer’s disease [10,11]. The pathogenesis of periodontal diseases is mediated by the inflammatory response of the host to the bacteria in the subgingival plaque [12]. Endothelin-1 is an important mediator of vascular inflammation and has been shown to be modulated by vitamin C and other antioxidants [13,14]. The subgingival microbial ecology is complex and many different bacterial species, predominately anaerobic bacteria, can inhabit the periodontal pocket [12,15]. However, *Porphyromonas gingivalis* is considered a keystone pathogen in the initiation and progression of periodontal disease due to its high proteolytic activity [16].

Mouthrinses containing antimicrobial substances such as amine fluoride/stannous fluoride or CHX are used for oral hygiene to prevent tooth decay [17,18]. However, already early on CHX-resistant bacterial strains, mostly Gram-negative species such as *Escherichia coli*, *Proteus* spp. or *Klebsiella* spp., have been described. Additionally, there is evidence that the extensive use of CHX could lead to the selection of strains being not only resistant to CHX but also to multiple antibiotics [19,20,21,22]. In a recent study analyzing the minimal inhibitory concentrations (MIC) of CHX and amine fluoride/stannous fluoride-containing mouthrinses against *P. gingivalis* and mutans streptococci, slightly elevated MIC values could be detected for CHX after long-term subinhibitory exposure for some of the *P. gingivalis* isolates only [23].

Beside resistance mechanisms, bacterial populations have other means to respond to antimicrobial substances. Bacteria can be tolerant against high concentrations of antimicrobial substances, usually by slowing down bacterial growth or by inactivating essential bacterial cell functions. Additionally, phenotypic variants of the wild type strain, termed persister cells, can arise when a bacterial population is treated with a bactericidal substance resulting in a biphasic time-kill curve [24]. In *E. coli*, the periodic application of a high concentration of a bactericidal antibiotic in vitro has led to the selection of high-persister (hip) mutants [25,26] and clinical isolates of *Candida albicans* from patients who had been treated regularly with CHX have elevated levels of such persister cells [27]. The formation of persister cells in *S. mutans* biofilms can be induced by the dental monomer dimethylaminohexadecyl methacrylate or by CHX [28]. So far, the formation of persister cells in oral microorganisms has been noticed in biofilms only [28,29].

The proper use of antibiotics and antimicrobials such as CHX is vitally important, especially when these substances are used over a long period of time. However, patients may use mouthrinses containing antimicrobial substances over a longer period with biocide concentrations lower than necessary to inhibit bacterial growth completely. Such long-term use may not only result in bacterial isolates with altered susceptibility profiles [23], but may also lead to the development of tolerant and/or persistent bacteria. Therefore, the aim of this study was to analyze the potential influence of long-term exposure in subinhibitory concentrations of CHX on the emergence of tolerant and/or persistent cells in oral streptococci in an in vitro study.

## 2. Results

**Figure 1:**
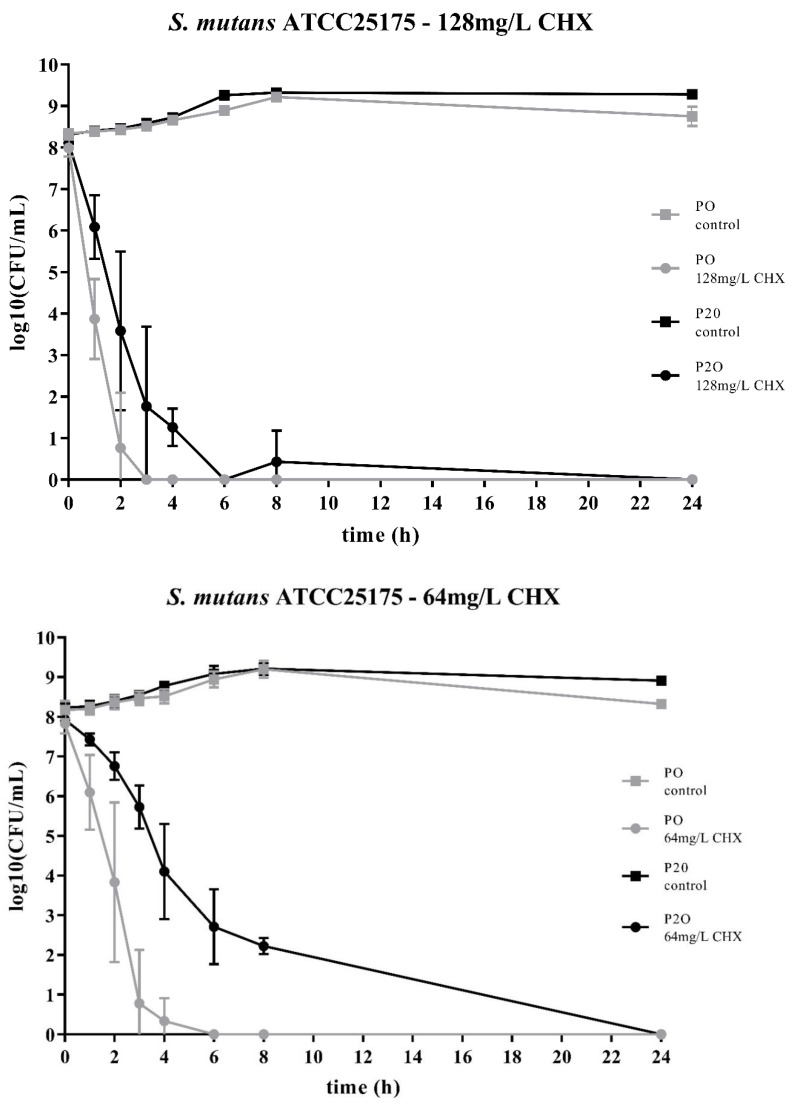
Time-kill curves of *S. mutans* ATCC25175 exposed to different concentrations of chlorhexidine (CHX). “P0” indicates the bacterial isolate that had not been passaged before on CHX, while “P20” denotes the isolate that have been subcultivated for 20 passages in subinhibitory concentrations of CHX, respectively. The streptococcal isolates were exposed to either 64mg/L or 128mg/L CHX. The values, shown as log10(CFU/mL), are means of three independent replicates. Error bars indicate the respective standard deviation. As control, untreated cultures without CHX were tested.

**Figure 2:**
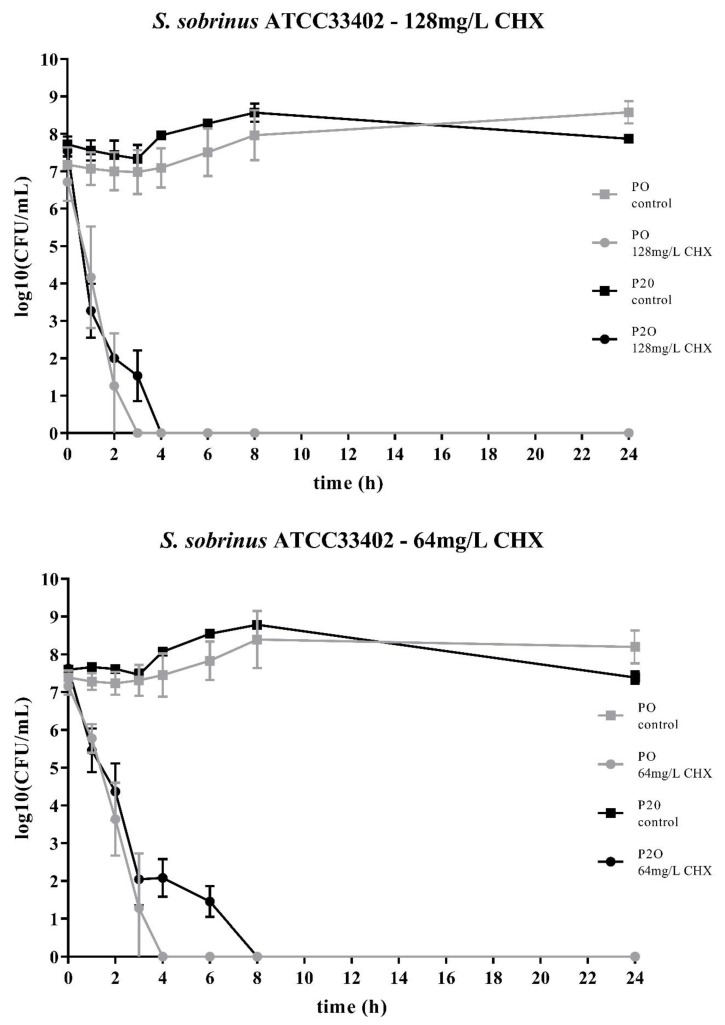
Time-kill curves of *S. sobrinus* ATCC33402 exposed to different concentrations of CHX. “P0” indicates the bacterial isolate that had not been passaged before on CHX, while “P20” denotes the isolate that have been subcultivated for 20 passages in subinhibitory concentrations of CHX, respectively. The streptococcal isolates were exposed to either 64mg/L or 128mg/L CHX. The values, shown as log10(CFU/mL), are means of three independent replicates. Error bars indicate the respective standard deviation. As control, untreated cultures without CHX were tested.

Two streptococcal strains were tested, i.e., *S. mutans* ATCC25175 and *S. sobrinus* ATCC33402. For both strains, isolates were compared that either had not been passaged on CHX (“P0” isolates) or that had been previously subcultivated for 20 passages in subinhibitory concentrations of CHX (“P20” isolates) [23]. For both streptococcal species, the decrease in bacterial cell counts was faster for the P0-isolates (Figure 1; Figure 2). At the concentration of 64mg/L CHX, no life counts could be detected after four to six hours for both P0-isolates. The decrease in bacterial growth was faster at the higher concentration of 128mg/L.

Accordingly, both streptococcal P20-isolates were more tolerant to CHX as it took more time to eliminate their growth. However, the time-kill curves were different for the two streptococcal species. For *S. sobrinus* ATCC33402, the treatment with CHX resulted in a biphasic time-kill curve, where an initial rapid decrease in bacterial counts was seen for both the P0- and the P20-isolate, with almost identical bacterial cell counts. After two to three hours, a distinct subpopulation of more CHX-tolerant cells could be detected. This subpopulation of potential persister cells was more pronounced at the lower CHX-concentration of 64mg/L (Figure 2).

The time-kill curves of the P20-isolates showed a different pattern for the other streptococcal species, S. mutans ATCC25175. For this streptococcal species, the P20-isolate appeared to be more tolerant to CHX than the P0-isolate, resulting in a generally slower decrease in bacterial cell counts over time (Figure 1).

## 3. Discussion

Both streptococcal isolates were tested with 64mg/L CHX and with 128mg/L CHX. As the respective MICs of CHX for these strains are between 0.5mg/L and 4mg/L, these concentrations are well above the MICs [23]. Accordingly, CHX was effective against all isolates and no live bacteria could be detected after 24 h. However, for both isolates and at both concentrations, the respective P20-isolates were more resistant to CHX and it took more time to eliminate their growth. The bacterial cell counts for all control suspensions, i.e., the bacterial cell suspensions that were not in contact with CHX, remained relatively constant over the period of time. However, the variability for the test suspensions, i.e., the bacterial cell suspension with added CHX, was, especially for the timepoints one to three hours, relatively large.

The two bacterial species showed different time-kill curves. Besides resistance, persister cell formation and tolerance are two other microbial survival strategies when treated with antibiotics or antimicrobial agents. The term ‘tolerance’ is used to describe the ability of microorganisms to survive the exposure to antimicrobial agents, in that the minimum duration for complete elimination is substantially higher than for a susceptible strain [24]. Such a situation could be noticed for *S. mutans* ATCC25175, where the P20-isolate appeared to be more tolerant to CHX compared to the P0-isolates as more bacterial cells were still alive at all timepoints. In contrast, the P20-isolate of *S. sobrinus* ATCC33402 showed a biphasic time-kill curve reminiscent of persister formation. Persister cells consist of a subpopulation of microorganisms that are capable to survive at higher concentrations of an antimicrobial agent. Typical biphasic time-kill curves are described by an initial exponential killing kinetics, indicating the susceptible population, and a second phase where the remaining bacteria are killed with a much slower kinetics [24,30,31]. In the case of the P20-isolate of *S. sobrinus* ATCC33402, this persister subpopulation consists of approximately 10^2^ cells per ml.

Antimicrobial resistance in bacteria is usually based on two major genetic strategies; mutations in target gene(s) or the acquisition of resistance conferring genes through horizontal gene transfer. Already early on, mechanisms not based on such genetic alterations have been noticed in clinical settings and later this phenomenon has been described in vitro as well [25,26,32]. In recent years, the ability of bacteria to temporarily survive high doses of antimicrobial substances has received increased attention, as many chronic diseases and recurrent episodes of infections appear to be associated with such resilient bacterial subpopulations [33,34].

Persister cells are phenotypic variants arising from a clonal population of genetically identical microbial cells. Different mechanisms have been described to explain how such persister cells arise. The formation of persister cells is often induced when bacterial cells are exposed to environmental stress or starvation. In our study, this stressor might have been the prior long-term incubation in subinhibitory concentration of CHX. In *S. mutans*, the competence-stimulating peptide CSP has been shown to act as a stress-inducible alarmone that can trigger an increased formation of multidrug-tolerant persister cells. Although the exact mechanisms are still unclear, one pathway may involve self-cleavage of the transcriptional repressor LexA [35]. For *Klebsiella pneumophila*, a Gram-negative opportunistic pathogen often resistant to multiple antibiotics, cross-resistances between CHX and colistin have been described [36]. The mechanisms of cross-resistance have been linked to mutations in efflux pumps and it has been suggested that a common mechanism exists for the adaptation of Enterobacteriaceae to different biocides [37]. As the Gram-positive streptococci are resistant to colistin, an antibiotic that is also used in streptococcal selective media, mechanisms might be different. Further studies on a molecular level are needed to pinpoint the involved adaptive mechanism in mutans streptococci. Besides environmental triggers, specific genes have been identified that are involved in persistence and when mutated, may increase the persister fraction within a bacterial population; examples include the *hip* locus, toxin-antitoxin modules and genes involved in SOS response [33,38,39].

Less is known on the presence of persister cells in oral microorganisms. *S. mutans* isolated from plaque samples of either caries-free children or of children with severe-early childhood caries (S-ECC), were tested for their ability to adapt to a lethal pH value and their survival rate after 24 h of ofloxacin treatment. *S. mutans* isolated from children with S-ECC showed an approximately 15-fold higher persistence phenotype [40]. Clinical isolates of *C. albicans* and *Candida glabrata*, which may cause invasive fungal infections in the oral cavity, were analyzed in cancer patients who had been treated daily prophylactically with CHX. High-persister strains could be isolated only from patients with *Candida* carriage of more than eight consecutive weeks but not from patients where *Candida* was present transiently. Additionally, persisters could also be detected in the presence of the antifungal drug amphotericin B [27]. In an in vitro study, persister cells could be observed *when C. albicans* biofilms were treated with CHX or the antifungal drug amphotericin B. Interestingly, this response could be detected only in biofilms but not in exponentially growing or stationary-phase planktonic cell cultures [29]. Similarly, persister cell formation by the dental monomer dimethylaminohexadecyl methacrylate or by CHX could be induced in *S. mutans* biofilms only [28]. This is in line with our study, where no persister cells were noticed for the P0-isolates tested in a planktonic culture assay.

However, long-term incubation in subinhibitory concentrations of CHX may lead to the emergence of a subpopulation of persister cells even when grown planktonically, as seen for the streptococcal P20-isolates. Subinhibitory concentrations of antimicrobial substances may also be present in the oral cavity where oral microorganisms are usually organized in biofilms. Biofilms form a protective habitat for bacterial cells that allows them to evade the immune response as well as the effect of antimicrobial substances and therefore, MIC values are higher for bacteria in biofilms than for planktonic cultures [41,42]. It has been suggested that persister cells may be a part of the explanation for the resilience of biofilms to antibiotics [42].

To control oral biofilms and as adjuvants for oral hygiene, mouthrinses containing antimicrobial substances, including CHX or amine fluoride/stannous fluoride, are commonly used. The cationic biguanidine CHX, introduced in 1954, is a widely used antimicrobial substance in dentistry and is considered to be the ‘gold standard’ of oral hygiene due to its ideal antimicrobial spectrum and remnant effect. However, adverse effects such as taste disturbance or tooth staining were noticed early on and allergic reactions including serious anaphylactic reactions have been described [18,43]. Although no clear definition for the term ‘resistance’ exists so far for antimicrobials such as CHX, intrinsic and acquired mechanisms conferring reduced susceptibility toward CHX have been described in various microorganisms as well as the potential risk of cross-resistance against other biocides or antibiotics [43].

The long-term use of subinhibitory concentrations of CHX may not only increase the MIC values of oral microorganisms, but might also lead to the induction of a more biocide-tolerant and persisting microbial subpopulation. Such mechanisms may also occur in the oral cavity; therefore, the rational and proper use of antimicrobials in dentistry, especially when used over a long period of time, is vitally important.

## 4. Materials and Methods

### 4.1. Bacterial Strains and Growth Conditions

Mutans streptococci tested were *S. mutans* ATCC25175 and *S. sobrinus* ATCC33402. “P0” indicates bacterial isolates that had not been passaged on CHX, while “P20” denotes isolates that have been subcultivated for 20 passages in subinhibitory concentrations of CHX, i.e., in a CHX concentration which was one serial dilution below the respective MICs. All isolates were stored in 20% glycerol at −80 °C [23].

Streptococcal P0-strains were maintained on Columbia agar (CA) plates (BBL; Becton Dickinson, Allschwil, Switzerland), while the respective P20-isolates were spread on Columbia agar plates supplemented with 0.25mg/L CHX (CA-CHX). This CHX-concentration is below the respective MIC-values and allow for the maintenance of CHX-tolerant bacteria. All streptococcal isolates were incubated in 10% CO_2_+air for 2–3 days.

### 4.2. Test Substance

Chlorhexidine digluconate (CHX) solution 20% was from Sigma-Aldrich Chemie GmbH (Buchs, Switzerland). A 0.2% CHX stock solution was prepared in H_2_O and further serially diluted in Columbia Broth (CB).

### 4.3. Testing of Mutans Streptococci

The mutans streptococci grown on the respective agar plates were resuspended in CB to a density of OD600 = 0.2 (approx. 1 × 10^8^ CFU/mL). The cultures were vortexed for one minute, ultrasonicated for ten seconds (6W; Vibracell, Sonics and Materials, Newtown, CT, USA) and divided into two parts. One was not treated (control suspension) and the other one was exposed to CHX (test suspension). Both cultures were again vortexed, split into 10 test tubes each and incubated aerobically at 37 °C. At predefined time points, one test tube each from the control suspension and one from the test suspension was vortexed for one minute, ultrasonicated for ten seconds (6 W; Vibracell, Sonics and Materials, Newtown, CT, USA) and the viable bacterial cell count was determined by plating 100 μL of appropriate dilutions onto CA plates.

### 4.4. Statistical Analysis

Analyses including mean and standard deviations were performed in GraphPad Prism 7.01.

## 5. Conclusions

Persister cell formation can be detected in streptococci that have been exposed to subinhibitory concentrations of CHX in vitro. Therefore, the rational and proper use not only of antibiotics but also of antimicrobials such as CHX is vitally important, especially when these substances are used over a long period of time.
